# Long-term survival and mortality predictors in a Swedish cohort of patients with ANCA-positive vasculitis and severe kidney involvement

**DOI:** 10.1093/ckj/sfag159

**Published:** 2026-05-25

**Authors:** Klytaimnistra Voudouri, Ylva Östlund, Karlo Mihovilovic, Daniel Kitamura Bylund, Maria K Svensson, Aso Saeed

**Affiliations:** Department of Medical Sciences, Renal Medicine, Uppsala University, Uppsala, Sweden; Department of Molecular and Clinical Medicine/Nephrology, Institute of Medicine, Sahlgrenska Academy, University of Gothenburg, Gothenburg, Sweden; Department of Molecular and Clinical Medicine/Nephrology, Institute of Medicine, Sahlgrenska Academy, University of Gothenburg, Gothenburg, Sweden; Department of Molecular and Clinical Medicine/Nephrology, Institute of Medicine, Sahlgrenska Academy, University of Gothenburg, Gothenburg, Sweden; Department of Medical Sciences, Renal Medicine, Uppsala University, Uppsala, Sweden; Uppsala Clinical Research Center, Uppsala, Sweden; Department of Molecular and Clinical Medicine/Nephrology, Institute of Medicine, Sahlgrenska Academy, University of Gothenburg, Gothenburg, Sweden

**Keywords:** albumin, albuminuria, ANCA-associated vasculitis, autoimmune diseases, kidney involvement, mortality, prognosis

## Abstract

**Introduction:**

To describe the clinical characteristics of a Swedish cohort of patients with anti-neutrophil cytoplasmic antibody (ANCA)-associated vasculitides (AAV) and kidney involvement and to study long-term survival and predictors of all-cause mortality.

**Methods:**

An observational study including patients with AAV and kidney involvement between 1993 and 2023 from two centres in mid-Sweden. Data were obtained from electronic health care records and follow-up visits. Multivariable Cox regression models and Kaplan-Meier analysis were performed to evaluate risk factors of interest predicting all-cause mortality.

**Results:**

Among 190 patients (56% men, median age 67 years, 43% PR3-ANCA (Proteinase 3 Anti-Neutrophil Cytoplasmic Antibody), and 57% MPO -ANCA (Myeloperoxidase Anti-Neutrophil Cytoplasmic Antibody) positive, estimated glomerular filtration rate at diagnosis was 23 ml/min/1.73 m^2^ [interquartile range (IQR) 11–42] and 16% required dialysis. Median follow-up 20 was 7.5 years (IQR 3.6–12.3) and 106 deaths occurred. Main causes of death were infections (28%), cardiovascular disease (24%) and malignancy (18%). Cox hazard regression models showed that older age [hazard ratio (HR) 1.10, 95% CI 1.06–1.14] was a strong, consistent, and independent predictor of all-cause mortality, irrespective of sex, ANCA type, and kidney function. In the Uppsala cohort (*n* = 91) serum albumin levels at diagnosis emerged also as an independent risk factor when adjusting for age, kidney function and other inflammatory parameters (HR 0.86, 95% CI 0.79–0.94).

**Conclusion:**

Long-term patient and kidney survival was associated mainly with age and serum albumin levels at diagnosis in this Swedish cohort of patients with AAV. Infections and cardiovascular disease were the most common causes of death, underscoring the importance of infection monitoring and cardiovascular risk management.

## Graphical Abstract

**Graphical Abstract:**
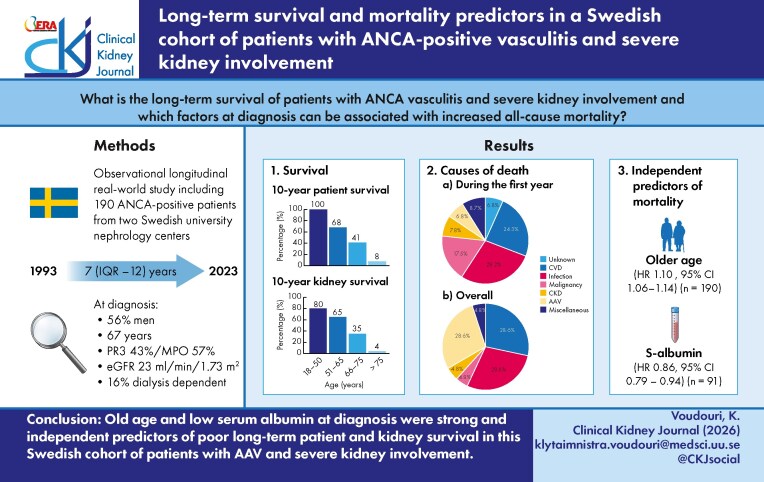

KEY LEARNING POINTS **What was known:**

- ANCA-associated vasculitis (AAV) is a life-threatening autoimmune disease that frequently leads to irreversible kidney damage.
- Despite advances in immunosuppressive therapy, selecting the optimal treatment remains a challenge given the heterogeneous patient characteristics, the risk of treatment-related complications and the potential for disease progression, such as irreversible kidney failure.

**This study adds:**

- This study includes patients with severe kidney impairment at diagnosis and shows that infections and cardiovascular events are leading causes of death.
- Older age and lower serum albumin independently predict poorer survival.
- PR3-ANCA positivity is associated with greater inflammatory burden and higher relapse rates, although survival is similar to MPO-ANCA–positive patients.

**Potential impact:**

- The findings in this study reinforce the importance of early disease detection and risk stratification in patients with AAV and kidney involvement, enabling clinicians to individualize immunosuppressive treatment.
- Additionally, early monitoring for infections and proactive management of cardiovascular risk factors may improve long-term outcomes in this high-risk population.

## INTRODUCTION

Anti-neutrophil cytoplasmic antibody (ANCA)-associated vasculitis (AAV) is a small-vessel vasculitis that can present at any age, with incidence of 17–20 per million person-years [1] and increasing prevalence of 300–421 per million persons, as a result of improved survival and diagnostic criteria [2–4]. The incidence peaks in the seventh and eighth decades, with elderly patients being particularly susceptible to the adverse effects of both the disease and its immunosuppressive treatment [4]. Despite treatment advancements, AAV patients still have considerable morbidity and mortality [5, 6]. Treatment complications and progress of disease remain significant contributors to reduced survival, with infections being the leading cause of mortality [7].

Kidney involvement is diagnosed in up to 78% in patients with granulomatosis with polyangiitis (GPA) and nearly 100% in microscopic polyangiitis (MPA) [3, 8]. The classic presentation involves rapidly progressive kidney disease characterized by crescentic glomerulonephritis and it is associated with significant morbidity and mortality [6]. The ANCA serotypes are shown to better discriminate between genetic associations, therapeutic response, relapse risk, prognosis, and co-morbidities [9]. Poor outcomes in AAV with kidney involvement are attributed to factors such as delayed diagnosis, inadequate treatment response, and long-term complications from both disease progression and side effects of immunosuppressive agents [5, 10–12]. In patients with AAV, the three main causes of death are infections, cardiovascular disease, and malignancies [13–15].

Previous studies have identified older age and male sex as independent predictors of poor survival [15, 16]. Recent population-based studies have reported increased mortality in patients with MPA, but no increase in patients with GPA compared to the general population [17].

The objective of this study was to evaluate the clinical and biochemical characteristics of a Swedish cohort of patients with AAV and severe kidney involvement at diagnosis. Additionally, it aimed to examine differences between ANCA-positive AAV in terms of treatment choices, long-term survival, and predictors of all-cause mortality.

## MATERIALS AND METHODS

### Patients

This observational cohort study initially included 197 patients diagnosed with GPA and MPA from two university nephrology clinics in mid-Sweden; Uppsala (*n* = 93) and Gothenburg (*n* = 104). Patients that were either ANCA-negative or double positive were then excluded (*n* = 7), resulting in a final cohort of 190 patients with MPO-ANCA-positive or PR3-ANCA-positive AAV and kidney involvement diagnosed between 1993 and 2023.

### Methods

Patients were identified using the ICD-10 codes M31.1-7. MPO-ANCA or PR3-ANCA-positivity was then verified via review of electronic healthcare records (EHRs). Clinical and laboratory variables, treatments and outcomes were obtained both from EHRs and at follow-up visits. Laboratory analyses were performed at the Departments of Clinical Chemistry at Uppsala University Hospital and Sahlgrenska University Hospital using routine methods. At Uppsala University Hospital testing of ANCAs was performed with the ELISA test until September 2008 and thereafter with ALBIA (addressable laser bead immunoassay) technique [18] and at Sahlgrenska University Hospital ANCA subtypes were analysed with capture-ELISA at the immunological laboratory.

Estimated glomerular filtration rate (eGFR) was calculated using creatinine-based 2021 CKD-EPI (Chronic Kidney Disease Epidemiology Collaboration) formula [19]. For two patients with missing creatinine values prior to the initiation of dialysis at diagnosis, the eGFR was set to 0 ml/min/1.73 m². Relapse was defined as the recurrence and/or appearance of one or more new vasculitis manifestations after remission identified from EHRs [20].

All study procedures were performed in accordance with the principles of the Declaration of Helsinki, and all study participants alive provided written informed consent prior to data collection. The study protocol was approved by the Swedish Ethical Review Board (Uppsala dnr 2021-05006) and by the regional ethics committee in Gothenburg (Sahlgrenska dnr 232-18).

### Statistical methods

Baseline clinical and biochemical characteristics were summarized using descriptive statistics. Normally distributed variables are presented as means ± standard deviation (SD), non-normally distributed variables as medians [interquartile range (IQR)], and categorical variables as frequencies (*n*) and percentages (%). Group differences were assessed using independent *t*-tests for normally distributed continuous variables, Pearson’s Chi-square test for categorical variables, and the Mann-Whitney *U* test for non-normally distributed continuous data. Log-transformation (Log^10^) was applied to C-reactive protein (CRP) and U-ACR values to reduce skewness. Spearman correlation coefficients were calculated to examine linear relationships. Survival data were measured in months from study start to death or end of study. Kaplan-Meier method and log-rank test was used to compare survival between groups. Cox proportional hazards regression analysis was used to evaluate the association between baseline characteristics and all-cause mortality, with the number of variables adjusted to the number of deaths (*n* = 106), maintaining a 10:1 event-to-variable ratio. All tests were two-tailed with significance set at *P* < .05. Analyses were conducted using IBM SPSS Statistics, version 28.0.

Regression tree analysis was performed using SurvCART in the R package LongCART.

## RESULTS

### Clinical and biochemical characteristics

Although some minor differences between the two centres were noted (Table S1), they were not considered significant enough to preclude pooling of the data. Clinical and biochemical characteristics of patients at the time of diagnosis are described in Table 1. Out of 190 patients, 109 (57.4%) were MPO-ANCA and 81 (42.6%) PR3-ANCA positive. The median age at diagnosis was 67 years (IQR 58–74). Patients with PR3-ANCA were significantly more likely to require dialysis and presented with higher levels of the inflammatory markers at diagnosis, including white blood cell count (WBC), platelets (PLT), and CRP. In the Uppsala cohort, patients with PR3-ANCA positivity had lower serum albumin levels (*P* = .05).

**Table 1:** Clinical and biochemical characteristics of patients with ANCA-positive vasculitis and kidney involvement at the time of diagnosis (*n* = 190).

Variables	Total (*n* = 190)	MPO-ANCA pos (*n* = 109)	PR3-ANCA pos (*n* = 81)	*P*-value
Sex M/F *n* (%)	113 (60)/77 (40)	59 (54)/50 (46)	54 (67)/27 (33)	.08
Age (years)	67 (58–74)	68 (59–74)	66 (52–73)	.09
Dialysis at diagnosis *n* (%) (*n* = 186)	29 (16)	12 (11)	17 (22)	.05
Smoking *n* (%) (*n* = 179)	28 (16)	16 (15)	12 (16)	.96
Pulmonary haemorrhage *n* (%) (*n* = 180)	23 (13)	10 (9.5)	13 (17)	.12
SBT (mmHg) (*n* = 161)	145 ± 22	147 ± 24	143 ± 18.5	.50
DBT (mmHg) (*n* = 161)	80 ± 12	81 ± 11	79 ± 11	.22
Creatinine (µmol/l) (*n* = 176)	220 (144- 416)	214 (151- 393)	238 (140–456)	.74
eGFR (ml/min/1.73 m^2^)* (*n* = 180)	23 (11–43)	22.6 (11.4–39.1)	24.5 (9.5–50. 5)	.8
S-albumin (g/l) (*n* = 69)**	27.4 ± 6.7	29 ± 6.3	23.5 ± 6.5	.05
Hb (g/l) (*n* = 146)	106 (94–117)	108 (96–117)	104 (92–118)	.4
WBC (× 10^9^/l) (*n* = 133)	9.2 (7.3–11.6)	8.3 (6.8–11)	10.5 (7.9–13)	.004
PLT (× 10^9^/l) (*n* = 133)	339 (282–411)	325.5 (260–357)	369 (331–476)	<.001
CRP (mg/l) (*n* = 146)	73 (17.5–140)	31 (8-108)	110 (53–170)	<.001
U-ACR (mg/mmol) (*n* = 124)	77 (35–200)	106 (36–219)	54 (33–153)	.074
Follow-up (yrs)	7.5 (3.6–12.3)	7.5 (3.5–11.5)	7.2 (3.8–13.6)	.79

Data are median (IQR) or mean ± sd or proportions *n*, %.

SBP; systolic blood pressure. Hb; haemoglobin, WBC; white blood cell count, PLT; platelets, CRP; C-reactive protein; U-ACR; urine albumin-creatinine ratio, IQR; interquartile range.

*eGFR, estimated glomerular filtration rate, estimated using creatinine-based 2021 CKD-EPI (19), eGFR for patients requiring dialysis at diagnosis was set to 5 0 ml/min/1.73 m^2^ if creatinine was not available (see methods section).

**S-albumin was only available from the Uppsala cohort (*n* = 91).

### Follow-up on treatments and outcomes

Follow-up data on treatments and outcomes are described in Table 2. The median follow-up time to death or end of study was 7.5 years (IQR 3.6–12.3), Induction therapy with cyclophosphamide (CYC) was more frequent in PR3-ANCA positive patients compared to MPO-ANCA positive patients (92 vs 73%, *P* < .001). Similarly, plasmapheresis (PLEX) was more frequently administered in the PR3-ANCA group (46 vs 29%, *P* < .01). Relapse of disease occurred more frequently in PR3-ANCA positive patients (47 vs 32%; *P* = .050). Notably, PLEX did not significantly impact kidney or patient survival, even in patients with severe kidney impairment (CKD 4–5) at diagnosis (Table S3). No difference was found between patients treated with CYC, rituximab (RTX) or PLEX as induction treatment in relation to developing serious infections requiring hospitalization during the first year from diagnosis (Table S4). Altogether, 106 deaths (56%) occurred during the follow-up period. The primary causes of death were infections (28%), cardiovascular disease (24%), and malignancy (18%). Infections (28%) and cardiovascular disease (28%) were the leading causes of death within the first year (Table S5).

**Table 2:** Follow-up data on treatments and outcomes in patients with ANCA-positive vasculitis and kidney involvement at diagnosis (*n* = 190).

Variables	Total (*n* = 190)	MPO-ANCA pos (*n* = 109)	PR3-ANCA pos (*n* = 81)	*P*-value
Induction CYC, *n* (%) (*n* = 186)	151 (8)	79 (73)	72 (92)	.001
Induction RTX, *n* (%) (*n* = 187)	34 (18)	16 (15)	18 (23)	.16
Induction PLEX, *n* (%) (*n* = 187)	68 (36)	31 (29)	37 (46)	.015
Relapse*, *n* (%) (*n* = 168)	62 (37)	33 (32)	29 (47)	.05
ESKD, *n* (%)	43 (23)	26 (24)	17 (21)	.60
Death, *n* (%)	106 (56)	62 (57)	44 (54)	.77
Hospitalized infections during the first-year, *n* (%) (*n* = 162)	56 (37)	32 (35)	24 (34)	.94
Pneumocystis infections during the first-year, *n* (%) (*n* = 167)	7 (4)	4 (4)	3 (4)	.98
Malignancy, *n* (%) (*n* = 159)	59 (37)	33 (35)	26 (40)	.5

Data are median (IQR), mean ± sd or proportions *n*,%. CYC; cyclophosphamide, RTX; rituximab, PLEX; plasmapheresis, IQR; interquartile range, ESKD; end-stage kidney disease*relapse was defined as the recurrence and/or appearance of ≥ one new vasculitis manifestation (s) after remission identified in EHRs.

### Univariable bivariate correlations

All-cause mortality correlated positively with age, need for dialysis, systolic blood pressure, and albuminuria (U-ACR) (*r* = 0.407, *P* < .001; *r* = 0.147, *P* = .045; *r* = 0.172, *P* = .029; and *r* = 0.208, *P* = .021, respectively), and negatively with baseline eGFR and haemoglobin (Hb) levels (*r* = −0.326, *P* < .001 and *r* = −0.190, *P* = .022, respectively) at diagnosis (Table S2a). In the Uppsala cohort (*n* = 91), serum albumin at diagnosis was inversely associated with all-cause mortality (*r* = −0.443, *P* < .001) (Table S2b).

### Overall and kidney survival time

Median overall survival time was 11 years (95% CI 8.5–13.4) with 5- and 10-year patient survival of 76 and 54%, respectively (Fig. S2). Kidney survival, defined as a composite endpoint of death or progression to end-stage kidney disease (ESKD, requiring dialysis or transplantation), was 67% and 47% at 5 and 10 years, respectively (Fig. S3).

### Age, kidney function, and albuminuria as predictors of mortality

Age and indicators of kidney involvement severity at diagnosis, including level of eGFR, need for dialysis and degree of albuminuria, were evaluated in Cox regression models adjusted for sex and ANCA-type (Table 3). Age was consistently associated with increased all-cause mortality (HR per year increase 1.09–1.11, all *P* < .001) across all models. When studied separately, need for dialysis at diagnosis was associated with a >3-fold increase in mortality risk: (HR 3.32, 95% CI 1.61–4.51). Lower eGFR and higher albuminuria were each associated with all-cause mortality in separate models; a 1 ml/min/1.73 m² decrease in eGFR increased mortality risk by 2% and a 10-fold increase in U-ACR increased risk by 86%. When analysed together, the associations were attenuated (eGFR HR 0.99; U-ACR HR 1.34) with confidence intervals crossing unity due to collinearity (*r* = −0.483, *P* < .001; Table S2a) reflecting shared prognostic information on kidney disease severity. No significant interaction eGFR × albuminuria was observed (Table S6). Sex and ANCA type were not associated with mortality in any model. These findings remained unchanged when dialysis-dependent patients were excluded from the analysis (data not shown). Furthermore, kidney survival analyses in the whole cohort were consistent with the risk prediction models for all-cause mortality (data not shown).

**Table 3:** Cox hazard regression analysis examining the impact of age, estimated glomerular filtration rate, albuminuria and need for dialysis at diagnosis of AAV on all-cause mortality, adjusted for sex, and ANCA type (*n* = 190).

	Model 1	Model 2	Model 3	Model 4
Variables at diagnosis	HR (95% CI)	HR (95% CI)	HR (95% CI)	HR (95% CI)
Age (years)	1.10 (1.06–1.14)	1.10 (1.06–1.14)	1.11 (1.08–1.14)	1.11 (1.08–1.14)
Sex (man)	0.94 (0.52–1.69)	0.88 (0.50–1.56)	0.95 (0.62–1.45)	0.88 (0.59–1.35)
ANCA-type (MPO pos)	1.40 (0.75–2.64)	1.46 (0.78–2.73)	1.40 (0.92–2.13)	1.33 (0.86–1.95)
U-ACR (mg/mmol)	1.34 (0.67–2.70)	1.86 (1.09–3.14)		
eGFR (ml/min/1.73 m^2^)	0.99 (0.97–1.00)		0.98 (0.97–0.99)	
Need for dialysis (yes)				3.32 (1.61–4.51)

U-ACR log^10^ transformed.

### Serum albumin at diagnosis

Serum albumin data were available in the Uppsala cohort (Table). In the multivariable Cox regression model including age, eGFR, and inflammation markers, including serum albumin, haemoglobin and CRP; older age and lower baseline serum albumin were consistently and independently associated with higher all–cause mortality during follow–up. The two strongest models, including Hb and PLT, respectively are presented in Table 4. These findings were confirmed in sensitivity analyses including other factors such as sex, ANCA-type or WBC (data not shown).

**Table 4:** Cox regression for all-cause mortality examining the impact of serum albumin at diagnosis of AAV on all-cause mortality, adjusted for age, kidney function, Hb, and CRP levels in the Uppsala cohort (*n* = 91).

	Model 1	Model 2
Variables	HR (95% CI)	HR (95% CI)
Age (years)	1.065 (1.02–1.11)	1.07 (1.02–1.12)
eGFR (ml/min/1.73 m^2^)	0.99 (0.97–1.01)	0.99 (0.97–1.01)
S-albumin (g/l)	0.86 (0.79–0.94)	0.85 (0.78–0.94)
CRP (mg/l)	0.99 (0.99–1.00)	0.99 (0.99–1.00)
Hb (g/l)	1.00 (0.98–1.02)	–
PLT (× 10^9^/l)	–	1.00 (1.00–1.01)

### Age and CKD stage as predictors of survival (Kaplan-Meier curves)

**Figure 1:**
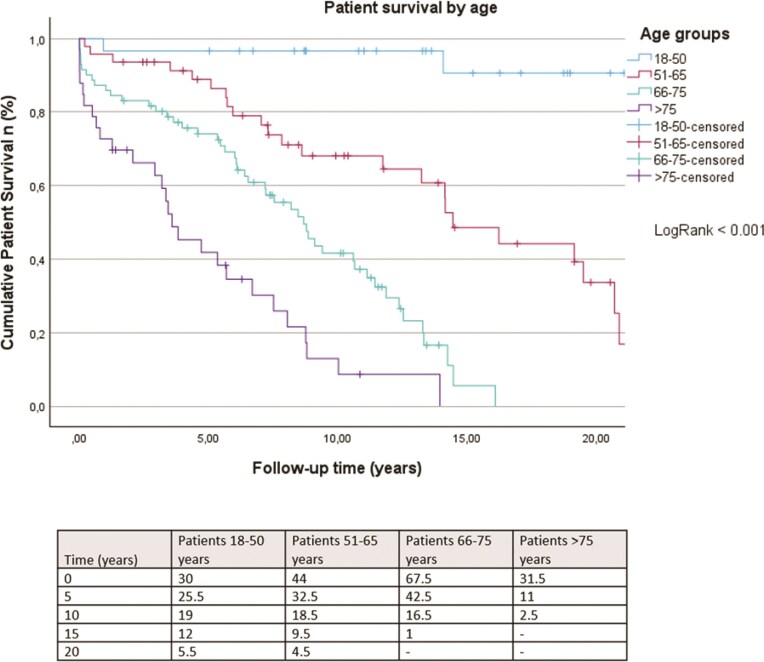
Patient survival by age-groups (18–50, 51–65, 66–75 and >75 years) in the overall cohort (n=190)

Kaplan-Meier analyses demonstrated that 10-year survival was 100% in patients <50 years, 68% in those aged 51–65 years, 42% in those 66–75 years, and 9% in patients >75 years. Survival curves separated early and widened over time, indicating both an immediate and sustained effect of age (Fig. 1).

Stratified by CKD stage, 10-year survival was 100% for patients <50 years and 75% for those 51–65 years with CKD stages 1–3, whereas patients 66–75 years showed an early, pronounced decline and those >75 years experienced rapid mortality even at early CKD stages (data not shown). In advanced CKD (stages 4–5), survival was markedly lower in patients 66–75 and >75 years (10-year survival 33% and 11%, respectively; data not shown). Mortality risk was particularly high for patients >75 years regardless of kidney function and for those aged 66–75 years with eGFR <30 ml/min/1.73 m².

**Figure 2:**
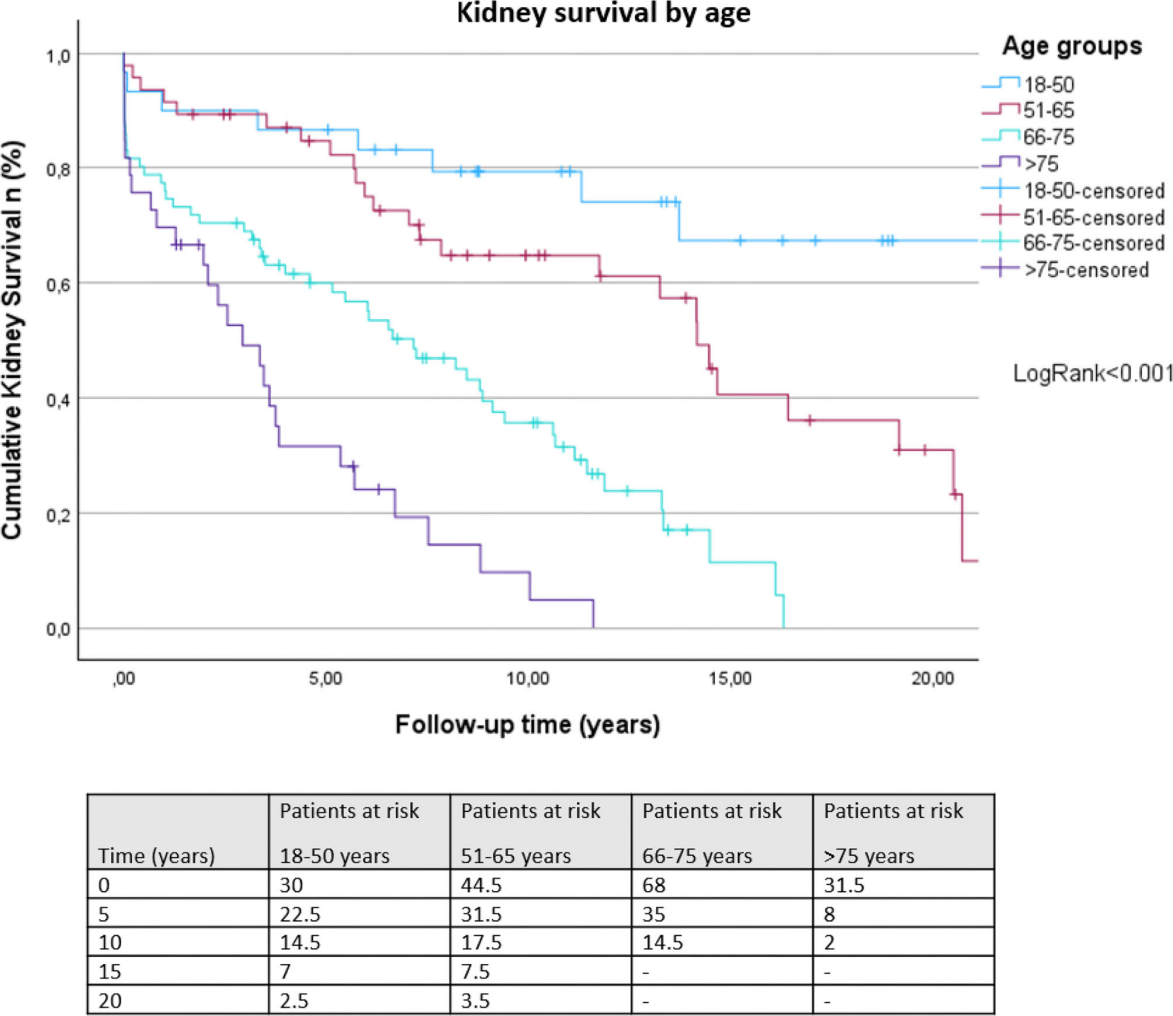
Kidney survival by age-groups (18–50, 51–65, 66–75 and >75 years) in the overall cohort (n=190).

Regression tree analysis supported the K-M curves identifying age >78 years associated with markedly higher mortality, independent of kidney function. Among patients <78 years, those aged 62–<78 years with eGFR <28 ml/min/1.73 m² had higher mortality (Fig. S1).

## DISCUSSION

In this observational study, we describe a Swedish cohort of patients with ANCA-positive vasculitis and kidney involvement at diagnosis, focusing on differences between PR3-ANCA and MPO-ANCA positive vasculitis in terms of treatment choices, long-term survival, and predictors of all-cause mortality. The overall mortality rate was high, 56% of patients died during the follow-up period with a median survival time of 11 years (95% CI 8.4–14.5). Mortality risk increased with age and worsening kidney function. Patients over 75 or 78 years, depending on analysis method, had the poorest survival, regardless of kidney function, while younger patients with severe CKD (stage 4–5 or eGFR <28 ml/min/1.73 m²) had higher mortality, with 10-year survival as low as 33% in those aged 62–78 years. Overall, older age combined with advanced CKD was associated with particularly poor survival outcomes, showing a combined effect of age and disease severity.

Infection was the leading cause of death, closely followed by cardiovascular disease and malignancies. These results underscore the importance of infection monitoring and cardiovascular risk management. In this study there was no comparison to the general population, but according to the 2023 rapport of the National Board of Health, the leading causes of death in the Swedish population were diseases of the circulatory system followed by malignancies (Statistics on Causes of Death 2023). The high infection-related mortality suggests that immunosuppressive therapies, while necessary, predisposes patients to severe complications. Our findings are in line with results from the French Vasculitis Study Group, which reported similar infection-related mortality rates in AAV cohorts [21]. Four patients in our cohort died from COVID-19 infection.

Median kidney survival time was 9 years (CI 95% 6.5–11.7) and gradually decreased with increasing age. Ten-year kidney survival for patients aged 18–50 years at diagnosis was 80% and decreased to 35% for patients aged 66–75 years. These findings are lower than those reported in previous studies, including the EUVAS trial, which reported similar survival estimates in patients with severe AAV [5, 15, 16]. The lower cumulative kidney survival observed in our cohort may be explained by selection bias since all patients were referred to a nephrology clinic with a higher proportion of patients requiring dialysis at diagnosis than reported in other studies [12]. No difference in long-term survival was found between PR3-ANCA positive and MPO-ANCA positive patients.

Patients with PR3-ANCA positivity had more severe inflammatory profiles at diagnosis, including higher levels of WBC, PTL, and CRP. Moreover, PR3-ANCA positive patients were more likely to require dialysis. All these findings align with previous studies, indicating that PR3-ANCA positivity is associated with more severe disease presentation with increased likelihood of more severe kidney and pulmonary involvement at onset, and increased relapse rates compared to MPO-ANCA positive patients [22–24].

Induction therapy with CYC was more common in PR3-ANCA positive patients, as was treatment with PLEX, which likely reflects the more aggressive disease course in these patients. Despite more intensive treatment strategies, PR3-ANCA positive patients exhibited a higher relapse rate compared to MPO-ANCA positive patients, consistent with previous studies that have demonstrated that PR3-ANCA positive patients had nearly double the relapse rates compared to MPO-ANCA patients [23, 24]. Interestingly, there was no difference in long-term survival between PR3-ANCA and MPO-ANCA patients in this study. Overall, the choice of treatment adhered to clinical guidelines.

Cox regression identified older age as independently associated with all-cause mortality. Reduced eGFR and increased albuminuria were each associated with all-cause mortality, in separate models though their effects were attenuated when included in the same multivariable model. This likely reflects that they share prognostic information, as eGFR and albuminuria both are markers of kidney disease severity, albuminuria reflecting ongoing glomerular and vascular injury dysfunction and eGFR cumulative loss of kidney function. Taken together, these findings demonstrate that age remains a strong, independent and important predictor of mortality, regardless of disease severity.

These findings align with prior studies indicating that kidney function at diagnosis is a determinant of long-term survival [12, 16], underlining the importance of early diagnosis of AAV, and especially with kidney involvement. To our knowledge, there is one previous study that has reported albuminuria and haematuria as prognostic factors of death and kidney survival after induction therapy and remission in AAV patients [25] and our study also identifies albuminuria as a clinically relevant risk factor. We find this particularly important as albuminuria is a well-recognized biomarker not only for progression of kidney disease, but also for chronic inflammation and risk of cardiovascular disease and death [26]. Unfortunately, reliable information on haematuria was not available in our study.

Serum albumin at baseline emerged as a strong and independent predictor of all-cause mortality after adjustment for age, kidney function and other markers of systemic inflammation while PLT at baseline were borderline significant in the Uppsala cohort. Low levels of serum albumin were strongly associated with all-cause mortality, with an effect size exceeding age, reflecting its role as a marker of disease severity and systemic inflammation in AAV. Nevertheless, this association cannot be attributed to inflammation alone, as other systemic inflammatory markers, except possibly for PLT, were not significantly associated with mortality. Low serum albumin also reflects additional pathophysiological processes, including albuminuria and the severity of kidney involvement. The robustness of the association between serum albumin and mortality across sensitivity analysis suggests that serum albumin underscores its potential as a simple, available, and clinically useful marker of mortality risk.

Age, baseline kidney function (eGFR), albuminuria, and serum albumin levels were evaluated as predictors of kidney survival, and the same variables associated with all-cause mortality were associated with kidney survival. This overlap was expected, as about two-thirds of the patients died before reaching ESKD, making mortality a major competing outcome.

Previous studies have also shown that male sex predicts a shorter long-term survival [16, 27], but this was not found in our study. Similarly, we did not find that other inflammatory markers at diagnosis, except possibly for PLT, were associated with all-cause mortality. Low PLT at baseline have previously been reported to be associated with survival [16].

PLEX did not significantly impact patient or kidney survival, even in patients with severe kidney impairment (GFR ≤30 ml/min/1.73 m²) at diagnosis, challenging the routine use in severe AAV. In line with our findings, the PEXIVAS trial demonstrated no significant survival benefit of PLEX in severe renal involvement [28], whereas earlier studies suggested a positive effect [29], possibly due to modern, standardized induction treatment protocols. Nonetheless, PLEX may still be considered in selected circumstances [30–32].

### Strengths and limitations

This study’s strengths include a well-characterized cohort with long-term follow-up, comprehensive clinical and biochemical data, and validated AAV diagnoses in all patients. Notably, 60% (108) had severe kidney impairment (CKD 4–5) at diagnosis, allowing detailed evaluation of this subgroup, unlike other studies [10, 12, 15, 16, 21, 25].

Limitations include a sample size that may limit subgroup and mortality predictor analyses, a cohort from a single country reducing generalizability, and changes in treatment guidelines during follow-up.

Future research should refine risk stratification using clinical and biochemical predictors, evaluate emerging therapies including targeted biologics, and prioritize strategies for early kidney protection to reduce progression to ESKD.

In conclusion, this study of Swedish AAV patients with kidney involvement shows that PR3-ANCA is linked to more aggressive disease and higher relapse, though survival was similar to MPO-positive patients. Older age and low serum albumin were strong and independent predictors of all-cause mortality, with infections and cardiovascular disease as leading causes of death. Findings highlight the need for early detection, individualized treatment, particularly taking age into account, and ongoing monitoring to improve outcomes.

## Supplementary Material

sfag159_Supplemental_Files

## Data Availability

The data underlying this article will be shared on reasonable request to the corresponding author.
