# Supplementary material for: Long-term survival and mortality predictors in a Swedish cohort of patients with ANCA-positive vasculitis and severe kidney involvement
Source: Clin Kidney J. 2026 May 25;19(7):sfag159. doi: 10.1093/ckj/sfag159 (PMC13339951; doi:10.1093/ckj/sfag159)
Supplement: sfag159_Supplemental_Files [file sfag159_supplemental_files.zip › Supplementary material.docx]

**Supplementary materials**

**Table S1.** Differences between centres in baseline characteristics, treatments, follow-up data, and outcomes.

| Variables | Uppsala (n=91) | Sahlgrenska (n=99) | P-value |
| --- | --- | --- | --- |
| Male/Female n(%) | 48 (53)/43 (47) | 65 (66)/34 (34) | 0.07 |
| MPO-ANCA/PR3 -ANCA n (%) | 68 (75)/23 (25) | 41 (41)/58 (59) | **<0.001** |
| Age (years) | 68 (60-74) | 66 (54-73) | **0.035** |
| Dialysis n (%)/total n | 16 (18)/91 | 13 (13)/95 | 0.46 |
| Smoking n (%) /total n | 10(11)/89 | 18 (18)/ 90 | 0.10 |
| Pulmonary haemorrhage n (%) | 11 (12)/89 | 12(12)/91 | 0.86 |
| SBT (mmHg) | 146±22/74 | 145±22/87 | 0.55 |
| DBT (mmHg) | 79.3±11/74 | 81±12/87 | 0.41 |
| Creatinine (µmol/L) (n=176) | 235 (161-391)/82 | 202 (129-438)/94 | 0.31 |
| eGFR (ml/min/1.73m^2^)* (n=180) | 21 (11-36)/86 | 29 (10-56.5)/94 | 0.07 |
| Hb (g/L) (n=146) | 104.5 (94-115) /74 | 108.5 (94-122)/72 | 0.17 |
| WBC (x 109 /L) (n=133) | 9 (7-12)/70 | 9 (8-12) /63 | 0.51 |
| PLT (x 109/L) (n=133) | 332 (283-387)/70 | 352 (277-425) /63 | 0.28 |
| CRP (mg/L) (n=145) | 65 (11.5-138)/73 | 78 (21-150)/73 | 0.26 |
| U-ACR (mg/mmol) (n=122) | 82 (35-218) /70 | 58.5 (33-162)/54 | 0.38 |
| Follow-up (years) | 5.7 (2.7-10.8) | 9 (6-13.6)/99 | **<0.001** |
| Induction CYC n (%) /total | 62 (68)/89 | 89(90)/97 | **<0.001** |
| Induction RTX n (%)/total | 13 (14)/89 | 21(21)/98 | 0.22 |
| Induction PLEX n (%) | 38 (42)/90 | 30(30)/97 | 0.10 |
| Relapse n (%) (n=168)** | 30 (33)/90 | 32(32)/76 | 0.24 |
| ESKD n (%) | 21(23)/91 | 22 (22)/99 | 0.88 |
| Death n (%) | 54 (60)/91 | 52(52.5)/99 | 0.34 |
| Time to dialysis (months) | 30 (0-76) | 32.5 (3-98) | 0.67 |
| Time to relapse (years) | 2.5 (1.8-6) | 2 (1-4) | 0.28 |
| Time to death (years) | 5 (0.8-10) | 6 (2-11) | 0.44 |
| Hospitalized infections during the first-year n (%) (n=162) | 20 (25)/80 | 36 (36)/82 | **0.01** |
| Pneumocystis infections during the first-year n (%) | 3 (4)/80 | 4 (4)/87 | 0.78 |
| Malignancy n (%)  (n=159) | 25 (28)/91 | 34 (34)/68 | **0.004** |

^Data are median (IQR) or mean±sd or proportions n, %. IQR; interquartile range. SBP; Systolic blood pressure. DBP; Diastolic blood pressure, Hb; Haemoglobin, WBC; White blood cell count, PLT; Platelets, CRP; C-Reactive Protein, U-ACR; Urine albumin-creatinine ratio, IQR; interquartile range, *eGFR; estimated glomerular filtration rate, estimated using creatinine-based CKD-EPI (Chronic Kidney Disease Epidemiology Collaboration equation (19), eGFR for patients requiring dialysis at diagnosis was set to 5 ml/min/1.73m2 if creatinine was not available (see methods section), CYC; Cyclophosphamide, RTX; Rituximab, PLEX; plasmapheresis, ESKD; end-stage kidney disease. *’Relapse was defined as the recurrence and/or appearance of ≥one new vasculitis manifestation(s) after remission identified in electronic health records (EHRs).^

**Table** **S2a.** Univariable bivariate correlations between baseline clinical and biochemical characteristics and all-cause mortality in patients with ANCA-positive vasculitis and kidney involvement at diagnosis (n=190)

|  | Age (years) | Sex | ANCA | Smoking | PH | Dialysis | SBP | DBP | eGFR | Hb | WBC | PLT | *CRP | *U-ACR |
| --- | --- | --- | --- | --- | --- | --- | --- | --- | --- | --- | --- | --- | --- | --- |
| Sex (men) | -.069  .346 |  |  |  |  |  |  |  |  |  |  |  |  |  |
| ANCA-type (MPO) | -.123  .092 | .126  .082 |  |  |  |  |  |  |  |  |  |  |  |  |
| Smoking (yes) (n=179) | .120  .109 | .114  .128 | **-.351^**^**  **<.001** |  |  |  |  |  |  |  |  |  |  |  |
| Dialysis at diagnosis (yes)  (n=186) | .090  .222 | .116  .115 | .140  .056 | -.001  .987 |  |  |  |  |  |  |  |  |  |  |
| Pulmonary haemorrhage (PH) (yes) (n=180) | -.087  .246 | .117  .118 | .115  .123 | -.108  .158 |  | **.259^**^**  **<.001** |  |  |  |  |  |  |  |  |
| SBP (mmHg) (n=161) | .146  .065 | -.037  .642 | -.053  .502 | -.057  .479 | .062  .435 | .123  .122 |  |  |  |  |  |  |  |  |
| DBP (mmHg) (n=161) | **-.166^*^**  **.035** | .082  .299 | -.050  .527 | -.005  .946 | -.029  .721 | -.011  .891 | **.575^**^**  **<.001** |  |  |  |  |  |  |  |
| eGFR at diagnosis (n=180) | **-.211^**^**  **.005** | .0.13  .866 | .021  .777 | -.070  .361 | -.036  .641 | **-.548^**^**  **<.001** | **-.292^**^**  **<.001** | -.081  .307 |  |  |  |  |  |  |
| Hb (g/L) (n=146) | **-.214^**^**  **.009** | **.189^*^**  **.022** | -.067  .420 | -.061  .470 | **-.262^**^**  **.002** | **-.341^**^**  **<.001** | -.069  .412 | **.197^*^**  **.017** | **.494^**^**  **<.001** |  |  |  |  |  |
| WBC (10^9^/L) (n=133) | -.005  .957 | -.001  .993 | **.254^**^**  **.003** | -.130  .140 | .125  .156 | **.192^*^**  **.027** | .007  .935 | .060  .495 | -.140  .107 | .031  .722 |  |  |  |  |
| PLT (10^9^/L) (n=133) | -.052  .549 | -.006  .946 | **.359^**^**  **<.001** | -.130  .140 | .057  .520 | .157  .072 | .015  .868 | -.030  .732 | .089  .308 | -.072  .416 | **.439^**^**  **<.001** |  |  |  |
| *CRP (mg/L) (n=145) | .049  .562 | .111  .184 | **.383^**^**  **<.001** | **-.210^*^**  **.012** | **.234^**^**  **.005** | **.387^**^**  **<.001** | -.105  .210 | -.110  .187 | **-.192^*^**  **.020** | **-.221^**^**  **.009** | **.510^**^**  **<.001** | **.352^**^**  **<.001** |  |  |
| *U-ACR (mg/mmol) (n=122) | -.050  .584 | .092  .314 | -.160  .078 | .224^*^  .013 | .020  .827 | .199^*^  .029 | .148  .107 | .144  .119 | **-.483^**^**  **<.001** | -.232^*^  .012 | -.088  .351 | -.126  .180 | -.169  .069 |  |
| All-cause mortality (yes) | **.407^**^**  **<.001** | -.001  .990 | -.025  .727 | .043  .567 | 0.089  2.37 | **.147^*^**  **.045** | **.172^*^**  **.029** | -.011  .889 | **-.326^**^**  **<.001** | **-.190^*^**  **.022** | .128  .141 | .135  .122 | .136  .103 | .**208^*^**  **.021** |
| Kidney survival | ,400^**^  <,001 | -,013  ,860 | -,019  ,792 | ,076  ,314 | 0.095  0.204 | ,250^**^  <,001 | ,185^*^  ,019 | ,003  0.97 | -,347^**^  <,001 | -,142  ,088 | ,179^*^  ,040 | ,116  ,183 | ,156  ,059 | ,253^**^  ,005 |

^PH; Pulmonary haemorrhage, SBP; Systolic blood pressure, DBP; diastolic blood pressure, Hb; Haemoglobin, WBC; White blood cell count, PLT; Platelets, CRP; C-Reactive Protein; U-ACR; Urine albumin-creatinine ratio, *eGFR was estimated using creatinine-based 2021 CKD-EPI (19)^

Table S2b. Univariable bivariate correlations between baseline clinical and biochemical characteristics and all-cause mortality in patients with ANCA-positive vasculitis and kidney involvement at diagnosis in the Uppsala cohort (n=91)

|  | Age (years) | Sex | ANCA | Smoking | PH | Dialysis | PH | SBP | DBP | eGFR | Hb | WBC | PLT | *CRP | *U-ACR | S-Albumin |
| --- | --- | --- | --- | --- | --- | --- | --- | --- | --- | --- | --- | --- | --- | --- | --- | --- |
| S-albumin (n=69) | -.215  0.077 | -.053  0.666 | **-.335**  **<0.005** | -.059  0.630 | -.210  0.086 | **-.426**  **<0.001** | -.210  0.086 | -.128  0.297 | .094  0.447 | **.400**  **<0.001** | **.256**  **<0.33** | **-.520**  **<0.001** | **-.427**  **<0.001** | **-.715**  **<0.001** | **-.005**  **0.972** |  |
| All-cause mortality (yes) | **.407^**^**  **<.001** | -.001  .990 | -.025  .727 | .043  .567 | -.089  .237 | **.147^*^**  **.045** | -.089  .237 | **.172^*^**  **.029** | -.011  .889 | **-.326^**^**  **<.001** | **-.190^*^**  **.022** | .128  .141 | .135  .122 | .136  .103 | .**208^*^**  **.021** | **-.443**  **<0.001** |
| Kidney Survival | ,260^*^  ,013 | -,006  ,958 | ,111  ,297 | ,118  ,270 | -,074  ,489 | ,280^**^  **0.007** | -,074b | ,140  ,233 | ,053  ,652 | -,260^*^  ,016 | -,092  ,436 | ,307^**^  ,010 | ,319^**^  ,007 | ,197  ,095 | ,163  0.185 | -,556^**^  <,001 |

^PH; Pulmonary haemorrhage, SBP; Systolic blood pressure, DBP; diastolic blood pressure, Hb; Haemoglobin, WBC; White blood cell count, PLT; Platelets, CRP; C-Reactive Protein; U-ACR; Urine albumin-creatinine ratio, *eGFR was estimated using creatinine-based 2021 CKD-EPI(19)^.

**Table S3.** Cox regression models for all-cause mortality and end stage kidney disease regarding the use of plasmapheresis as induction therapy in a subgroup of patients with CKD stage 4-5 or CKD 5 only

|  | Plasmapheresis (yes) | P-value |
| --- | --- | --- |
| CKD 4-5 (n=106) | n = 54 |  |
| Death from any cause | 1.18 (0.71-1.95) | 0.51 |
| End-stage kidney disease or death | 1.10 (0.58-2.10) | 0.75 |
|  | | |
| CKD 5 (n=66) | n = 40 |  |
| Death from any cause | 1.17 (0.72-1.90) | 0.50 |
| End-stage kidney disease or death | 1.03 (0.56-1.88) | 0.90 |

**Table S4** Difference between patients treated with Cyclophosphamide, Rituximab or plasmapheresis as induction treatment in relation to developing serious infections requiring hospitalisation during the first year from diagnosis

|  | Hospitalized infections in the first year | PCP infection in the first year |
| --- | --- | --- |
| Induction with RTX (n=34) | 11 (32.4), p= 0.73 | 3 (8.8), p=0.13 |
| Induction with CYC (n = 151) | 45 (29.8) p= 0.49  Missing cases 25 | 6 (4), p=0.67  Missing cases 20 |
| Induction with PLEX (n = 68) | 19 (27.9), p =0.99  Missing cases 13 | 3 (4.4), p=0.69  Missing cases 8 |

**Table S5.** Causes of death in the cohort (overall and during the first year after diagnosis)

| **Cause of death** | **Overall (n=103)** | **First year after diagnosis (n=21)** |
| --- | --- | --- |
| Infection | 29 (28.2%) | 6 (28.6%) |
| Cardiovascular Disease | 25 (24.3%) | 6 (28.6%) |
| Malignancy | 18 (17.5%) | 1 (4.8%) |
| Miscellaneous | 9 (8.7%) | 1 (4.8%) |
| Chronic Kidney Disease | 8 (7.8%) | 1 (4.8%) |
| ANCA-associated vasculitis | 7(6.8%) | 6 (28.6%) |
| Unknown | 7 (6.8%) | 0 |
| Missing | 3 | 1 |

**Table S6.** Cox regression analysis examining the impact of **interaction between albuminuria and kidney function** at diagnosis of AAV **on all-cause mortality (n = 190)**

| Variables | B | HR | 95% CI | P value |
| --- | --- | --- | --- | --- |
| **Age (years)** | **0.093** | **1.10** | **1.05 – 1.13** | **<0.001** |
| Sex (man) | -0.159 | 0.94 | 0.52 – 1.69 | 0.84 |
| ANCA-type (MPO pos) | 0.34 | 1.40 | 0.74 – 2.66 | 0.29 |
| U-ACR (mg/mmol) | 0.32 | 1.38 | 0.47 – 4.01 | 0.54 |
| eGFR (ml/min/1.73m2) | -0.012 | 0.98 | 0.94 – 1.03 | 0.58 |
| **eGFR (ml/min/1.73m2) x U-ACR (mg/mmol)** | **-0.001** | **0.99** | **0.97 – 1.02** | **0.93** |

**Table S7.** Cox regression analysis examining the impact of **kidney function (CKD stage 4-5) and albuminuria** at diagnosis of AAV on all-cause mortality, adjusted for age, sex, and ANCA type (n=190)

| Variables | B | HR | 95% CI | P value |
| --- | --- | --- | --- | --- |
| **Age (years)** | **0.10** | **1.10** | **1.06-1.14** | **<0.001** |
| Sex (man) | -0.05 | 0.95 | 0.53-1.69 | 0.86 |
| ANCA-type (MPO pos) | 0.39 | 1.48 | 0.70-2.78 | 0.22 |
| U-ACR (mg/mmol) | 0.28 | 1.33 | 0.70-2.51 | 0.38 |
| CKD-stage at diagnosis (CKD 4-5) | 0.67 | 1.95 | 0.91-4.15 | 0.08 |
