# Supplementary figures and images for: Long-term survival and mortality predictors in a Swedish cohort of patients with ANCA-positive vasculitis and severe kidney involvement
Source: Clin Kidney J. 2026 May 25;19(7):sfag159. doi: 10.1093/ckj/sfag159 (PMC13339951; doi:10.1093/ckj/sfag159)

## Slide 1
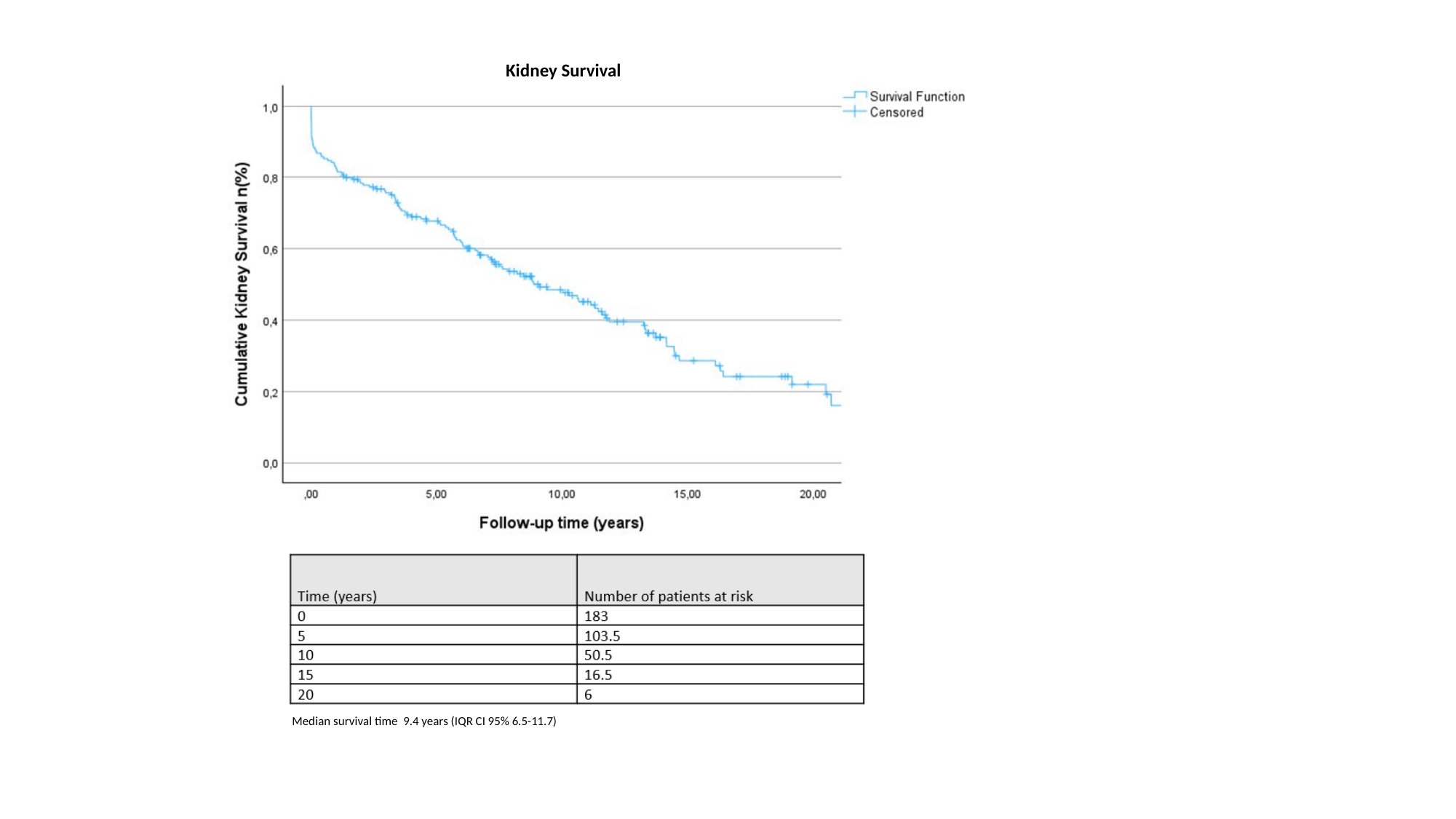

Kidney Survival
Median survival time 9.4 years (IQR CI 95% 6.5-11.7)

Supplement: sfag159_Supplemental_Files [file sfag159_supplemental_files.zip › Kidney Survival_supplementary file.pptx]

## Slide 1
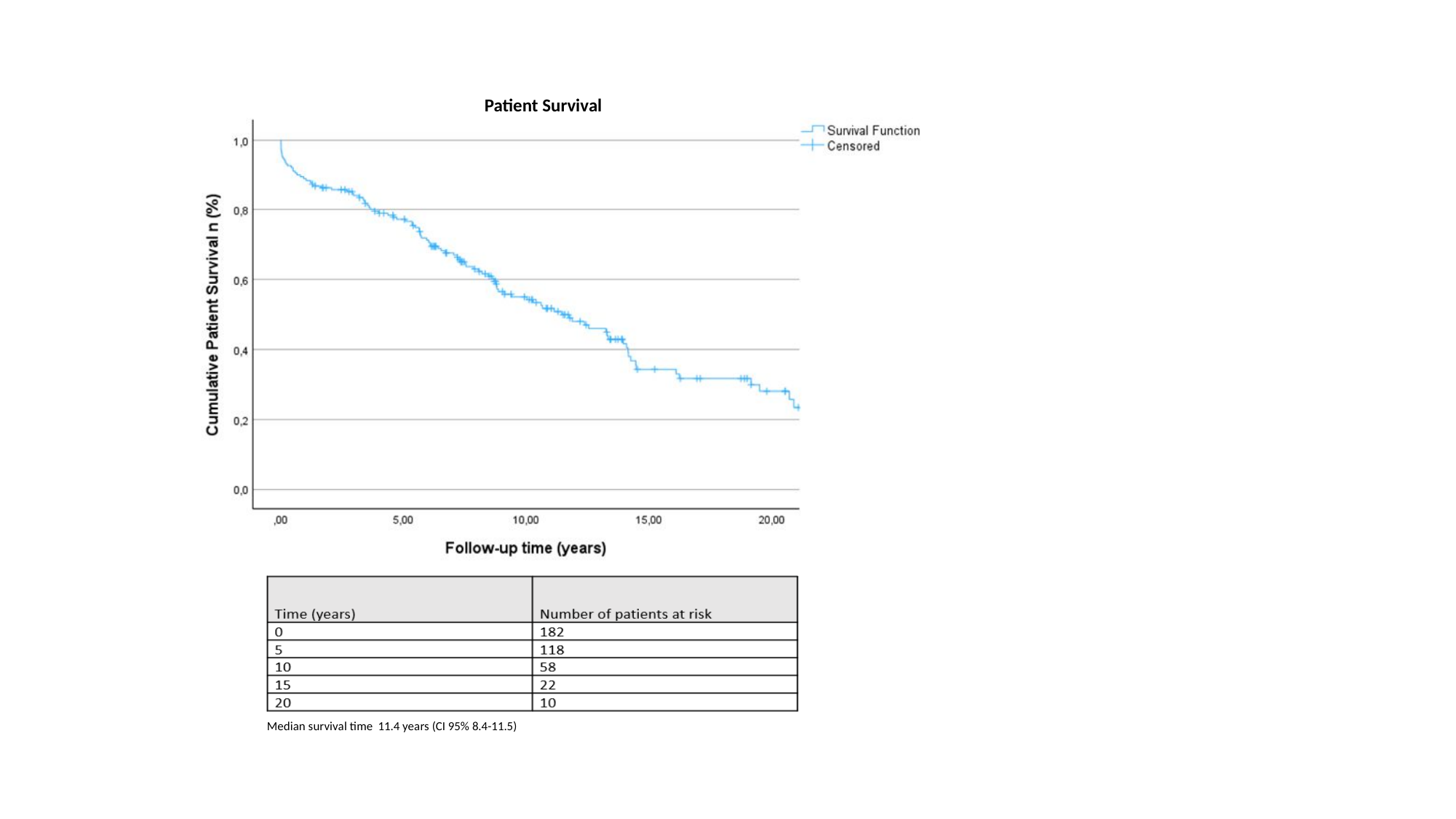

Patient Survival
Median survival time 11.4 years (CI 95% 8.4-11.5)

Supplement: sfag159_Supplemental_Files [file sfag159_supplemental_files.zip › Patient Survival_supplementary file.pptx]

## Slide 1
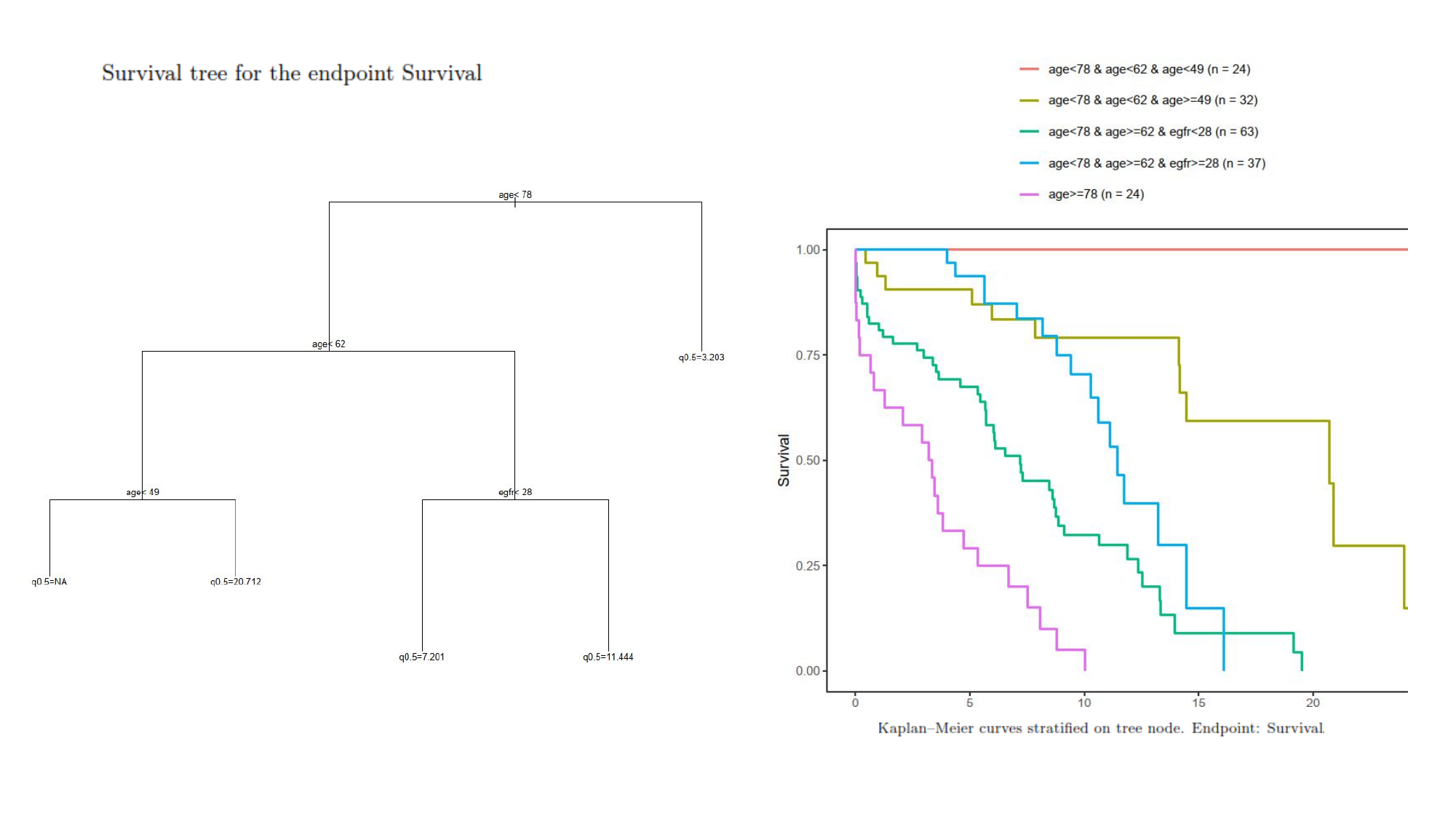

Supplement: sfag159_Supplemental_Files [file sfag159_supplemental_files.zip › Regression tree analysis_supplementary file.pptx]
